# Optimal male fertility and fecundity in *Caenorhabditis elegans* require Microprocessor and Argonaute gene function

**DOI:** 10.1093/g3journal/jkag079

**Published:** 2026-04-02

**Authors:** Lu Lu, Carmela Rios, Allison L Abbott

**Affiliations:** Department of Biological Sciences, Marquette University, Milwaukee, WI 53201, United States; Department of Biological Sciences, Marquette University, Milwaukee, WI 53201, United States; Department of Biological Sciences, Marquette University, Milwaukee, WI 53201, United States

**Keywords:** microRNA, Microprocessor, male, male fertility, sperm production, small RNAs, WormBase

## Abstract

Small RNA pathways play key roles in the regulation of gene expression in the germ line and in somatic cells. The germ line in *Caenorhabditis elegans* has multiple classes of small RNAs that can interact with its 19 Argonaute family proteins, including microRNAs (miRNAs), Piwi-interacting RNAs (piRNAs), and endogenous small interfering RNAs (endo-siRNAs). The pathway for miRNA biogenesis and activity requires stepwise processing before the mature miRNA is bound to an Argonaute protein and can function in a miRNA-induced silencing complex (miRISC). To understand whether the small RNA pathway responsible for producing miRNAs is required for optimal male fertility and fecundity, we analyzed male fertility, sperm production, and gonad morphology in mutants with reduced Microprocessor activity and miRNA-associated Argonaute activity. Optimal male fertility requires the Microprocessor genes, *drsh-1* and *pash-1*, as well as multiple miRNA-associated Argonaute genes. Although mutant male sperm display normal in vitro sperm activation, multiple mutants displayed defects in the ability to generate cross-progeny upon successful mating. Together, our results support a role for small RNA pathway genes in germline or somatic cells to promote optimal male fertility and fecundity.

## Introduction

The production of functional gametes is crucial for successful animal reproduction. Male factor infertility accounts for 30% to 50% of infertility cases and can be caused by a range of problems with sperm quality, quantity, or function (Boivin et al. 2007; Agarwal et al. 2015, 2021; Eisenberg et al. 2023). Sperm production is a dynamic process involving male germ cell proliferation and differentiation. In the male germ line, germ cells proliferate and progress into meiosis to produce motile sperm capable of fertilizing the oocyte (Gunes et al. 2018). Gene regulatory mechanisms are required for optimal sperm production, including post-transcriptional regulation by microRNAs (miRNAs) (Papaioannou and Nef 2010; McIver et al. 2012; Wu et al. 2012; Yadav and Kotaja 2014; Yao et al. 2015; Robles et al. 2017). miRNAs are enriched in spermatogenic cells and display distinct expression profiles at different stages of spermatogenesis (Yang et al. 2013; Liu et al. 2015). Clinical studies show that disrupted miRNA expression is associated with defects in sperm quantity or quality (Abu-Halima et al. 2013; Flannigan et al. 2017; Zhang et al. 2020; Abu-Halima et al. 2021), suggesting both positive and negative roles of miRNAs in this process. Furthermore, a subset of miRNAs that has been implicated to be necessary for normal sperm production may have potential to be prognostic biomarkers for sperm defects (Kotaja 2014; Hilz et al. 2016; Walker 2022; Graziani et al. 2024; Hosseini et al. 2024; Shi et al. 2024).

**Fig. 1.**
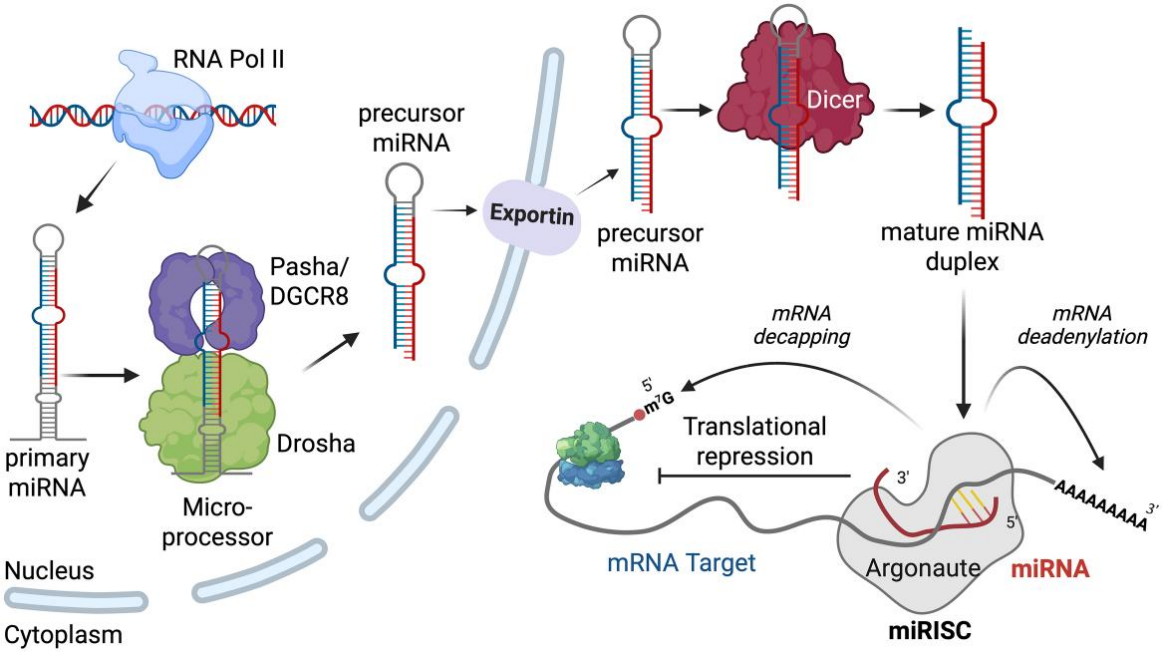
The miRNA biogenesis pathway. Briefly, miRNA genes are transcribed by RNA polymerase II (RNA Pol II). The primary miRNAs (pri-miRNAs) are then processed into precursor miRNAs (pre-miRNAs) in the nucleus by the Microprocessor complex, which includes the ribonuclease enzyme Drosha and the RNA-binding protein Pasha/DGCR8. After pre-miRNA export to the cytoplasm, Dicer further cleaves the pre-miRNAs into short mature miRNAs. Mature miRNAs associate with Argonaute proteins to form a miRNA-induced silencing complex (miRISC). The miRISC binds to target mRNAs with imperfect sequence complementarity, which can lead to translational repression and mRNA destabilization through decapping and deadenylation. Figure 1 was created in BioRender, https://BioRender.com/m9ioxl4.

miRNAs are short noncoding RNAs, around 22 nt in length, that typically mediate target gene regulation through translational repression and mRNA degradation (Kim et al. 2025). For most miRNAs, RNA polymerase II transcribes miRNA genes to generate a primary miRNA that is first processed by the Microprocessor complex, which is composed of Pasha/DGCR8 and Drosha proteins, to form a stem-loop precursor miRNA that is then processed by Dicer in the cytoplasm to form the mature miRNA (Fig. 1). Mature miRNAs interact with Argonaute proteins to form a miRISC that can bind to the 3′UTR of its targets and repress translation (Kim et al. 2025). In mice, loss of Drosha activity in spermatogenic cells results in decreased production of both pachytene spermatocytes and round spermatids (Wu et al. 2012). Additionally, loss of Pasha/DGCR8 activity in germ cells is associated with reduced sperm production and impaired sperm maturation (Zimmermann et al. 2014). While miRNA-mediated gene regulation is important for optimal male fertility, the precise mechanisms mediated by miRNAs are still largely unknown.

In *Caenorhabditis elegans*, miRNAs function to regulate diverse processes including developmental timing, aging, germline function, and embryonic development (Ambros and Ruvkun 2018; Kotagama and McJunkin 2024). *C. elegans* male and hermaphrodite gonads contain diverse miRNAs with distinct expression patterns in the germ line (McEwen et al. 2016; Minogue et al. 2018; Bezler et al. 2019; Lu and Abbott 2024), indicating possible roles in regulating meiotic progression and germ cell development. Germ cell proliferation and differentiation defects are observed in miRNA-associated Argonaute mutants (Bukhari et al. 2012; Conine et al. 2013; Brown et al. 2017). Microprocessor mutants and miRNA-associated Argonaute mutants display ovulation defects in hermaphrodites (Rios et al. 2017). Both the germline specific Argonaute *alg-5* and the *mir-44* family of miRNAs function to regulate sperm production in hermaphrodites (Brown et al. 2017; Maniates et al. 2021). In addition, a set of miRNAs have been shown to regulate meiotic progression and oocyte development (Minogue et al. 2018). Lastly, a set of miRNAs were found to be necessary for optimal sperm production and meiotic progression in the male germline (Lu and Abbott 2024). Since previous studies have mainly focused on hermaphrodites, the roles of Microprocessor and miRNA-associated Argonautes in regulating male fertility and fecundity are not well understood.

Here, we performed a set of assays to determine effects on male fertility and fecundity with the loss or reduction of Microprocessor or miRNA-associated Argonaute gene activity. We found that perturbation of the Pasha/DGCR8-Drosha Microprocessor complex or Argonaute function is associated with defective male fertility, germline development, sperm production, and sperm function. These analyses support potential regulatory roles for miRNAs in male reproduction and provide insights for further dissection of mechanisms that control male fertility, sperm production, and sperm function.

## Materials and methods

### Strain maintenance

All *C. elegans* strains were maintained on NGM plates seeded with AMA1004 (Casadaban et al. 1983). To facilitate sperm quantification in males, the *him-8(e1489)* mutation and a *Phis-72::HIS-72::GFP(stls10027)* integrated transgene were crossed into all Microprocessor or Argonaute mutant strains (Huang et al. 2012). This is referred to as “*him-8; his-72::gfp*” herein. A strain with *him-8 (e1489); his-72::gfp (stIs10027)* was used as a control in all experiments unless otherwise specified. Strains analyzed in this study are listed in Supplementary Table 1.

### Mating assays

Mating assays were performed by picking a single L4-stage male of control or mutant strains and an L4-stage CB933 (*unc-17(e245)*) hermaphrodite to an NGM plate with a smaller bacterial lawn compared to standard maintenance NGM plates. The following day (day 1), the male was removed, and the hermaphrodite was transferred to a new NGM plate every day until the end of its reproductive life. For mating assays at 15 °C, males and hermaphrodites interacted for approximately 1.5 d. Mating success was determined by the presence of non-Unc cross-progeny, and the number of cross-progeny and self-progeny was counted. The percent cross-progeny was calculated (the number of cross-progeny divided by total number of progeny).

### Sperm quantification

Sperm quantification in virgin males was performed as previously described (Huang et al. 2012; Lu and Abbott 2024). Briefly, a single male was picked into a 3 uL drop of sperm buffer (50 mM HEPES pH7, 25 mM KCL, 45 mM NaCl, 1 mM MgSO4, 5 mM CaCl2, 10 mM Dextrose pH7.8) on a cover glass, and a slide was gently placed on top to release the sperm without causing dispersal away from the male. Slides were then examined with epifluorescence microscopy using a Nikon 80i compound microscope with a 60× objective to directly count the number of GFP-positive (*his-72::gfp* transgene expression) haploid spermatids with their characteristic condensed chromatin. Microprocessor mutant males were picked at the early L4 stage and transferred to fresh plates to prevent mating. The number of sperm was scored in young adult males 10 h later. The Argonaute mutant males were counted at the L4 molt stage.

### Sperm activation

In vitro sperm activation was performed using pronase as previously described (Singaravelu et al. 2011). Males were washed with M9 and picked into a drop of fresh sperm buffer (50 mM HEPES pH7, 25 mM KCL, 45 mM NaCl, 1 mM MgSO4, 5 mM CaCl2, 10 mM Dextrose pH7.8) with pronase (20 µg/mL) on a cover glass. Spermatids were released by cutting the worm with 2 fine-gauge needles. After incubation with or without pronase, Vaseline was applied to the edges of the cover glass, and a slide was placed on top. Sperm were analyzed for activation using DIC microscopy with a 60× objective.

### Brood size

A single L4-stage hermaphrodite worm was picked and transferred to a new plate every day during its reproductive period. The number of progeny on each plate was counted, and the total number of progeny was calculated for each individual worm.

### RNA interference

Control, *him-8;his-72::gfp*, or mutant, *alg-2; him-8; his-72::gfp*, embryos were transferred from NGM plates to an RNAi plate seeded with HT115 bacteria with a control plasmid (L4440) or with a plasmid expressing dsRNA to knock down *alg-1* (Kamath and Ahringer 2003). Two days later, males were imaged using epifluorescence microscopy with a 40× objective.

### Fluorescence microscopy

Nomarski DIC and fluorescence microscopy were performed using a Nikon Eclipse Ti2 inverted microscope with a 40× objective (NA1.3). Images were acquired using an ORCA-Fusion BT camera (HAMAMATSU) with Z-stacking to capture the entire germline. Image analysis was performed using Nikon NIS-Elements software.

### Statistical analysis

The analysis between control and mutants was conducted with unpaired t-test or ANOVA followed by Dunnett, Dunnett's T3, or Tukey’ test using GraphPad Prism software. For all figures, the statistical difference is denoted as ns, *, **, ***, and ****, when *P* > 0.05, *P* < 0.05, *P* < 0.01, *P* < 0.001, and *P* < 0.0001, respectively. The details for each statistical analysis are included in the figure legends.

## Results

### Male fertility is dependent on functional Microprocessor

**Fig. 2.**
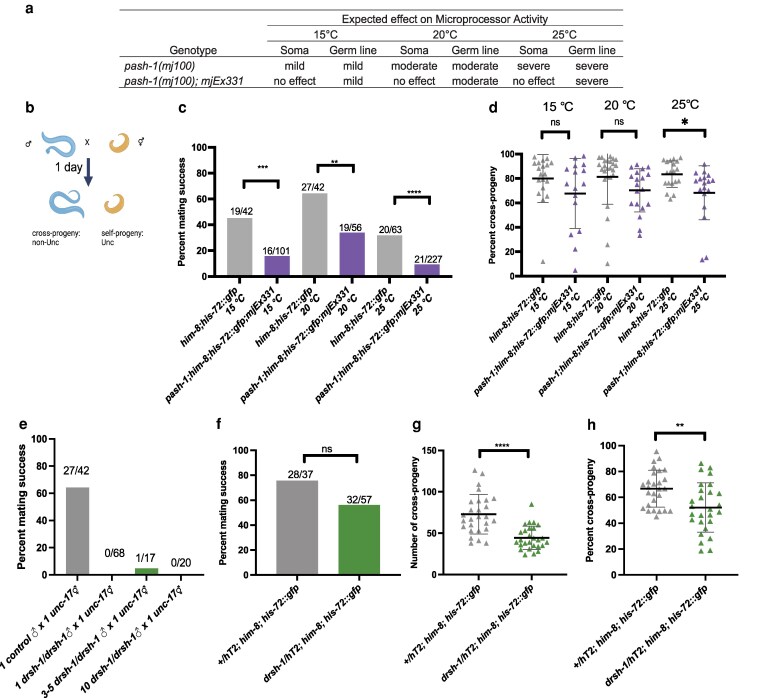
Male fertility is sensitive to miRNA biogenesis disruption due to defective Microprocessor activity. a) Expected effect on miRNA biogenesis caused by *pash-1(mj100)* temperature sensitive mutation with or without the *mjEx331* rescuing transgene extrachromosomal array in the soma and germline. b) Overview of the mating assay. c) The percentage of mating assays with cross-progeny sired by control (gray) or mutant males (purple). The number of successful and total mating assays is indicated above each bar. The comparison between control and mutant males was made using Fisher's exact test. d) The percentage of cross-progeny from successful matings by control (gray) or mutant male (purple). e) No successful matings were observed in mating assays with one or multiple *drsh-1* mutant males. The number of successful and total mating assays is indicated above the bar. f) Male mating efficiency of *drsh-1*/hT2 mutant was not different from controls. g) Lower number of cross-progeny was observed from matings with *drsh-1*/hT2 mutant males (green) relative to controls (gray). Comparisons were conducted by Welch's t-test. h) Lower percentage of cross-progeny was observed from matings with *drsh-1*/hT2 mutant males (green) relative to controls (gray). Each triangle represents the percentage d and h) or total number g) of cross-progeny from a successfully mated hermaphrodite and lines indicate mean ± SD. Unpaired t-tests were used to compare control and mutant results. The statistical analysis between control and mutant was represented with ns, *P* > 0.05; *P < 0.05; **P < 0.01; ***P < 0.001; and ****P < 0.0001. Details are included in Supplementary Table 2.

First, we focused on analyzing 2 genes that encode core proteins in the Microprocessor complex, *pash-1* and *drsh-1*, in *C. elegans*; *pash-1* encodes the Pasha/DGCR8 homolog while *drsh-1* encodes the Drosha homolog. The *mj100* allele is a temperature sensitive, loss of function *pash-1* allele that shows reduced activity at elevated temperature (Fig. 2a; Lehrbach et al. 2012). At 15 °C, *pash-1(mj100)* mutant worms develop normally and mature miRNAs are present (Lehrbach et al. 2012). However, at 25 °C, *pash-1(mj100)* mutants are not viable and have strongly reduced mature miRNA levels (Lehrbach et al. 2012). Mating assays conducted with Unc hermaphrodites and individual *pash-1* mutant males (Fig. 2b) showed no production of cross-progeny at 15 °C, 20 °C, or 25 °C (Supplementary Fig. 1a). Even with extended time to promote male-hermaphrodite interactions at 15 °C, there was still no cross-progeny observed (Supplementary Fig. 1a). This indicated that male fertility is strongly dependent on Microprocessor activity mediated by normal *pash-1* function in the soma or in the germ line.

To analyze disruption of *pash-1* activity in the germ line, we used a *pash-1(mj100)* strain with the *mjEx331* extrachromosomal array that expresses *pash-1(+)* controlled by the *eft-3* ubiquitous promoter (Lehrbach et al. 2012). Because extrachromosomal arrays are typically silenced in the germ line after a few generations (Kelly et al. 1997), the *pash-1(mj100); mjEx331* provides a genetic background with primarily somatic *pash-1(+)* activity at the restrictive temperature (Fig. 2a). These *pash-1(mj100); mjEx331* mutants are viable at 25 °C. Mating assays were performed with *pash-1; mjEx331* males at 15 °C, 20 °C, and 25 °C. Mutant males had reduced mating success compared to control males at all temperatures, with the strongest effect observed at 25 °C at which temperature only 9% of mutant males successfully produced cross-progeny compared to 32% of control males (Fig. 2c; Supplementary Table 2). These male fertility defects at all temperatures analyzed demonstrated that somatic expression of *pash-1(+)* by *mjEx331* was not sufficient to recover full male fertility, despite strong rescue observed for other somatic defects (Lehrbach et al. 2012). Hermaphrodites that were successfully mated with mutant males had a decreased percentage of cross-progeny at 25 °C, but not at 15 °C or 20 °C (Fig. 2d; Supplementary Table 2). When males mate with hermaphrodites, the male sperm is typically used preferentially to fertilize oocytes and hermaphrodites will produce cross-progeny until the male sperm supply is exhausted. Interestingly, mutant males had a comparable total number of cross-progeny relative to control males (Supplementary Fig. 1b), indicating that sperm transfer and sperm function were largely maintained in *pash-1(mj100)*; *mjEx331* mutant males.

We then analyzed male fertility and fecundity in *drsh-1* mutants. Because *drsh-1* mutant hermaphrodites are sterile, first-generation homozygous mutants were isolated from *drsh-1(ok369)/hT2* hermaphrodites. Male fertility was severely affected in *drsh-1* mutants. None of the *drsh-1* mutant males in our 68 mating assays produced cross-progeny (Fig. 2e). One successful mating was observed when mating assays were conducted with multiple males (Fig. 2e). For this individual mating event, it was observed to have 44.8% cross-progeny. To determine if *drsh-1* was haplo-insufficient, heterozygous *drsh-1/hT2* mutants were also analyzed. While mating efficiency was comparable to controls (Fig. 2f; Supplementary Table 2), matings with *drsh-1(ok369)/hT2* mutant males had fewer cross-progeny (Fig. 2g; Supplementary Table 2) with a decreased percentage of cross-progeny compared to what was observed in matings with *+/hT2* males (Fig. 2h; Supplementary Table 2). This could be caused by defects in sperm transfer or in sperm function. Together, these data suggest that optimal male fertility requires Microprocessor function. Further, both somatic and germline function of Microprocessor may contribute to the regulation of male fertility and fecundity.

### Optimal male sperm production requires Microprocessor activity

**Fig. 3.**
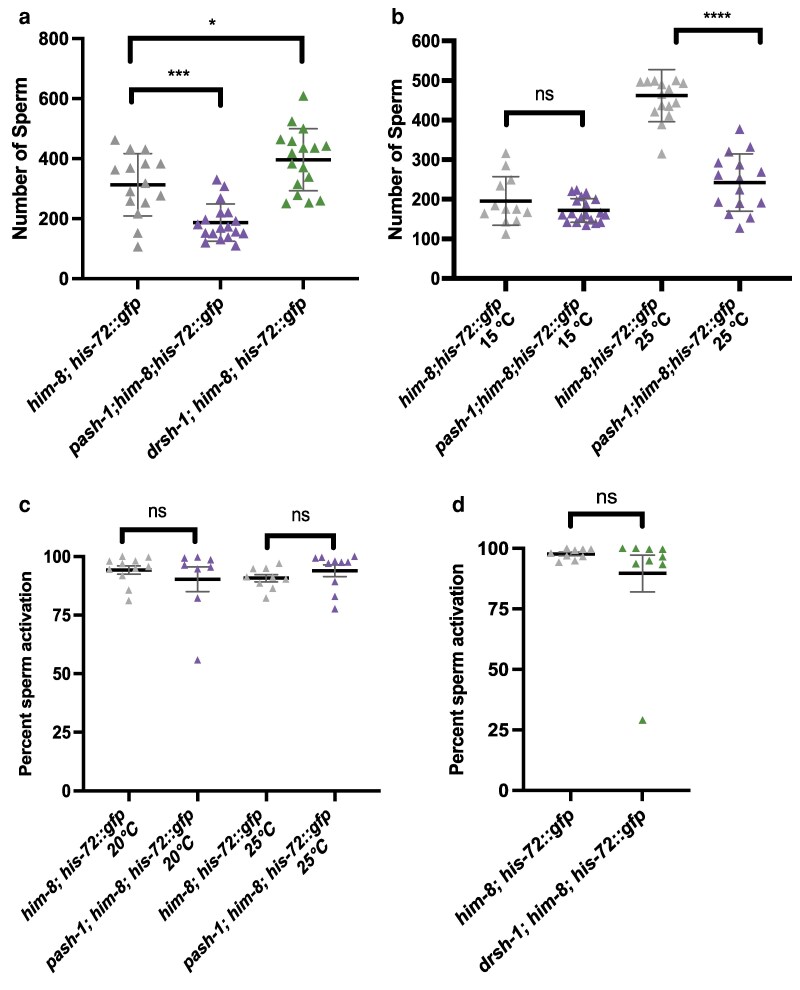
Microprocessor mutants had decreased male sperm production. a) Sperm quantification in *pash-1* (purple) or *drsh-1* (green) mutant males at 20 °C. The comparison between control (*him-8; his-72::gfp*) and mutants is shown above each data set from 1-way ANOVA followed by Dunnett's test. b) *pash-1* mutant males had reduced sperm production at 25 °C, but not at 15 °C. Mutant males were compared to control by Welch's t-test. c) In vitro sperm activation of *pash-1* mutant males was not different from control sperm activation at 20 °C or 25 °C. For analysis at 25 °C, early larval–stage *pash-1* mutant males were shifted from 15 °C to 25 °C. d) *drsh-1* mutants had normal in vitro sperm activation. Comparisons were conducted using Welch's t-test, and each triangle represents the number a and b) or percent activation c and d) of sperm per individual male and lines indicate mean ± SD a and b) or mean ± SEM c and d). The statistical analysis between control and mutant was represented with ns, *P* > 0.05; *P < 0.05; **P < 0.01; ***P < 0.001; and ****P < 0.0001. Details are included in Supplementary Table 3.

The male germ line produces sperm starting from the L4 stage and continuing throughout the reproductive lifespan of the adult. We first examined how reduced Microprocessor activity affects sperm production by counting the number of sperm in control and mutant young adult males. The number of sperm in *pash-1(mj100)* mutant males was not different from control males at 15 °C but was significantly decreased at both 20 °C and 25 °C (Fig. 3, a and b; Supplementary Table 3). Unlike *pash-1(mj100)* mutants, *drsh-1(ok369)* mutant males had slightly more sperm compared to control L4-stage males at 20 °C (Fig. 3a; Supplementary Table 3). These defects could reflect different levels of Microprocessor activity in these genetic mutant backgrounds or that miRNA biogenesis activity does not equally depend on *pash-1* and *drsh-1* activity.

### Spermiogenesis does not require Microprocessor activity

In vitro activation assays were performed to determine the ability of mutant male sperm to complete spermiogenesis. *C. elegans* male sperm become motile spermatozoa capable of migrating to the spermatheca and fertilizing oocytes after transfer to the hermaphrodite. Both *pash-1(mj100)* and *drsh-1* mutant male sperm showed comparable levels of sperm activation as control male sperm (Fig. 3, c and d), suggesting that sperm activation does not require Microprocessor activity in *C. elegans*.

### Male fertility is regulated by a subset of Argonaute proteins

In addition to the Microprocessor genes, *pash-1* and *drsh-1*, Argonaute genes are also essential for the biogenesis and function of small RNAs. Argonaute proteins are at the functional core of the small RNA-induced silencing complex (RISC) and are associated with diverse small RNAs including miRNAs, piRNAs, 22G-RNAs, and 26G-RNAs. There are 19 Argonaute proteins in *C. elegans*, and they are grouped in different clades (Seroussi et al. 2023): the Ago clade includes seven Argonautes: ALG-1, ALG-2, ALG-3, ALG-4, ALG-5, EGO-1, and RDE-1. ALG-1, ALG-2, and ALG-5 primarily associate with miRNAs, while ALG-3 and ALG-4 associate with both 26G-RNAs and miRNAs (Table 1). ALG-3 and ALG-4 are critical for sperm production and function under temperature stress. Here, we studied male fertility and sperm production in a subset of miRNA-associated Argonaute mutants (*alg-1*, *alg-2*, *alg-3*, *alg-4*, *alg-5*, and *rde-1*).

**Table 1.** Summary of Argonaute clade proteins in *C. elegans*^a^.

Argonaute protein	Argonaute-associated small RNAs^b^	Spatial expression in hermaphrodites^c^	Spatial expression in adult males
ALG-1	**miRNAs**	Soma	Soma
ALG-2	**miRNAs**	Soma Germline (adult only)	Soma
ALG-3	26G-RNAs **miRNAs**	Germline	Germline
ALG-4	26G-RNAs miRNAs	Germline	Germline
ALG-5	**miRNAs**	Germline	Germline^d^
RDE-1	**miRNAs** 22G-RNAs	Soma	Expressed^e^
ERGO-1	26G-RNAs miRNAs	Soma Germline (adult only)	Soma

^a^Association with small RNAs and Expression Data summary from Seroussi et al. (2023).

^b^The predominant type of small RNA is shown in bold.

^c^Only the expression during L4 and adulthood is indicated.

^d^The spatial expression is indicated from Brown et al. (2017).

^e^The spatial expression is not characterized in Seroussi et al. (2023).

**Fig. 4.**
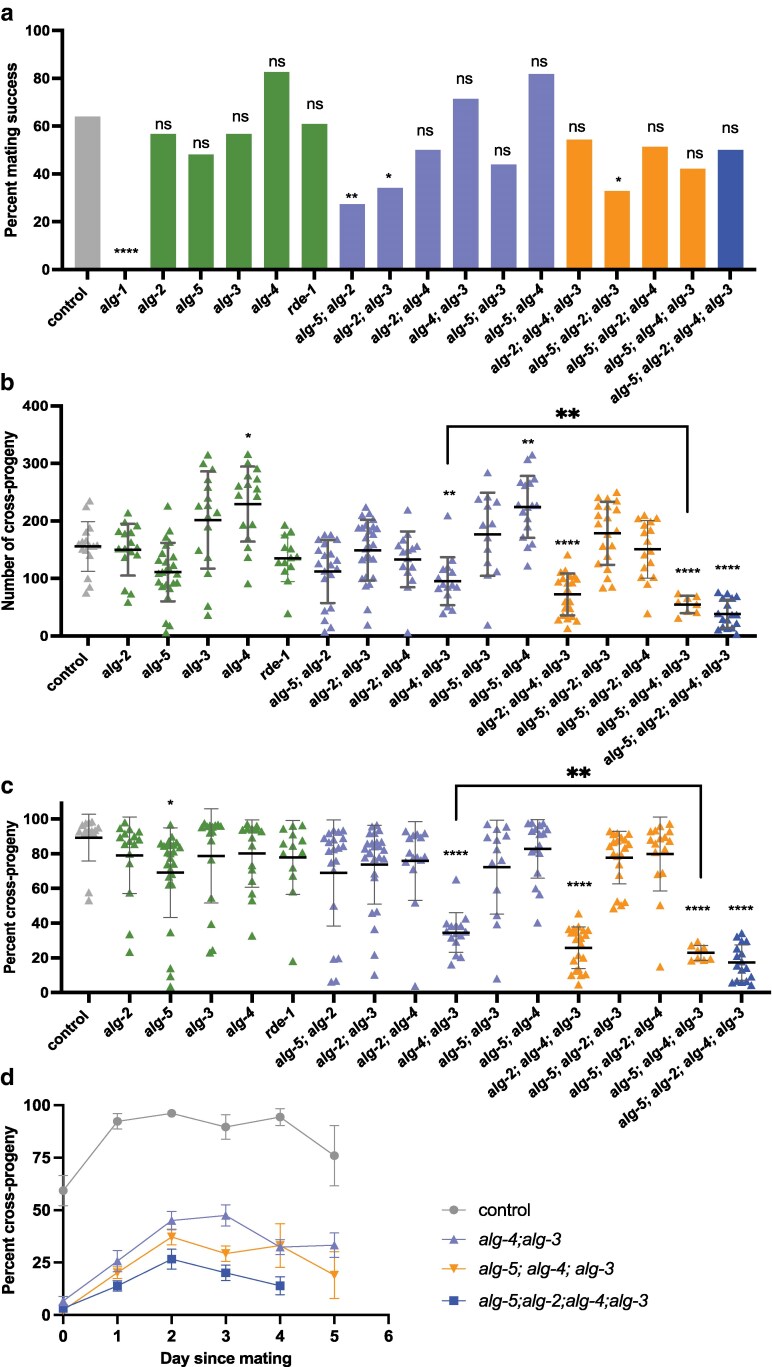
A subset of miRNA-associated Argonaute mutants displayed mating defects. Male mating assays were conducted for controls (gray), single (green), double (purple), triple (orange), and quadruple (blue) Argonaute mutants. a) Male mating efficiency was reduced in a subset of Argonaute mutants. The percentage of mating success is indicated by bars and *n* > 19 mating assays conducted per strain. The comparisons between control and mutants were conducted using Fisher's exact test. b) The number of cross-progeny was lower in multiple mutants. c) Percentage of cross-progeny was lower in multiple mutants. Brown–Forsythe ANOVA test followed by Dunnett's T3 was used to make comparisons between control and specific mutants. The comparison between *alg-4*;*alg-3* and *alg-5*;*alg-4*;*alg-3* was conducted using Welch's t-test. d) The percentage of cross-progeny quantified for each day following mating for a subset of control and mutants. The mutants consistently showed a significantly lower percentage of cross-progeny compared to controls on all days, as determined by multiple unpaired-t-tests. Lines indicate mean ± SEM. Each triangle represents the number b) or percentage c) of cross-progeny from a successfully mated hermaphrodite, and lines indicate mean ± SD. The statistical analysis between control and mutant was represented with ns, *P* > 0.05; *P < 0.05; **P < 0.01; ***P < 0.001; and ****P < 0.0001. Details are included in Supplementary Table 4.

Because it is possible that miRNA-associated Argonautes could function redundantly to affect male fertility, single mutants and multiple mutants were analyzed. First, *alg-1* mutant males failed to produce cross-progeny in our mating assays (*n* = 35), while other single Argonaute mutant males were comparable to control males in the percentage of mating assays that resulted in cross-progeny (Fig. 4a; Supplementary Table 4). Fewer *alg-5; alg-2* double mutant males produced cross-progeny, which was similar to what was observed in mating assays with *alg-2; alg-3* double mutant males. However, no further additive effect was observed in *alg-5; alg-2; alg-3* triple mutant males. This suggests that a shared pathway may be affected in the *alg-5; alg-2* and *alg-2; alg-3* double mutants. Because *alg-5* and *alg-3* are primarily expressed in germ line (Brown et al. 2017; Seroussi et al. 2023), these data are consistent with the observation from *pash-1* mutants that small RNA pathways in the germ line may be needed for optimal male fertility.

### Argonaute proteins may function together to control male sperm production and function

Fewer cross-progeny were produced for a subset of Argonaute mutants (Fig. 4b; Supplementary Table 4), despite successful matings with hermaphrodites (Fig. 4a; Supplementary Table 4). This was observed for *alg-4; alg-3* double mutants, *alg-2; alg-4; alg-3* and *alg-5*, *alg-4; alg-3* triple mutants, and the *alg-5; alg-2; alg-4; alg-3* quadruple mutant. Loss of *alg-3* and *alg-4* has been shown to cause defects in sperm production and sperm activation at 25 °C (Conine et al. 2010, 2013). Interestingly, loss of *alg-5* enhanced the defects in cross-progeny production associated with *alg-4*;*alg-3* mutants, with loss of *alg-2* resulting in no further reduction in cross-progeny in the quadruple mutant. The percentage of cross-progeny was also decreased in a subset of mutants with the *alg-5; alg-2; alg-4; alg-3* quadruple mutants displaying the lowest percentage of cross-progeny (Fig. 4c; Supplementary Table 4). Mutant male sperm exhibited a partial loss of competitiveness over hermaphrodite sperm in fertilizing oocytes as both self-progeny and cross-progeny were produced (Fig. 4d). The production of both cross-progeny and self-progeny suggests that either fewer sperm are transferred upon mating or that the advantage of mutant male sperm over hermaphrodite self-sperm was compromised.

**Fig. 5.**
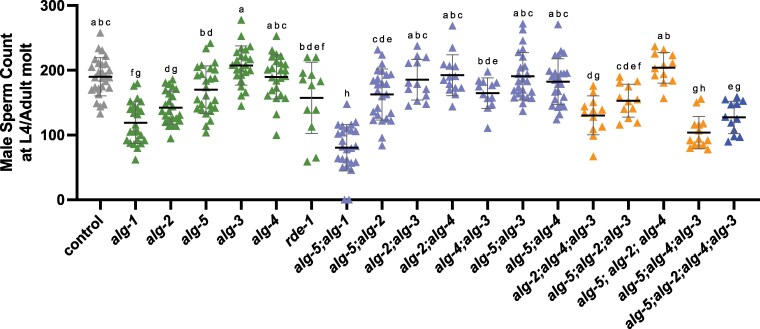
Genetic interaction between miRNA-associated Argonaute genes in regulating male sperm production. The number of sperm produced in males at the L4 molt stage was counted for control (gray), single (green), double (purple), triple (orange), and quadruple (blue) Argonaute mutants. Sperm quantification was performed using *his-72::gfp* to detect haploid spermatids. All strains had *him-8; his-72::gfp* in the background. Each triangle represents the number of sperm from a single male (*n* > 12 for each strain), and lines indicate mean ± SD. Comparisons were conducted using 1-way ANOVA followed by Tukey's post hoc test, and statistical differences are denoted by letters. Different letters indicate statistically significant differences (*P* < 0.05), whereas groups sharing a letter are not significantly different. Details are included in Supplementary Table 4.

We next examined sperm production in Argonaute mutants. *alg-1* and *alg-2* single mutant males produced fewer sperm than controls at the L4 molt stage. Sperm production in *rde-1* mutant males was moderately affected, showing an intermediate phenotype between *alg-2* mutant and control males. We observed a further reduction in the number of sperm in *alg-5; alg-1* double mutant males (Fig. 5; Supplementary Table 4). Therefore, we further examined the germline in the *alg-5; alg-1* and *rde-1* mutant males (Supplementary Fig. 2a). Whereas *rde-1* mutant males showed no observable defects in gonad morphology, smaller gonads were observed in *alg-5; alg-1* double mutant males, suggesting that fewer germ cells likely account for the reduced sperm production. Defects in germ cell proliferation and differentiation observed in *alg-1* and *alg-5* hermaphrodites (Bukhari et al. 2012; Brown et al. 2017) could potentially contribute to the mutant male sperm defects. The *alg-5; alg-4; alg-3* triple mutant males also had enhanced defects in sperm production relative to single and double mutants in these genes (Fig. 5; Supplementary Table 4), and this was not further enhanced by the loss of *alg-2* (Fig. 5; Supplementary Table 4). Together, these data indicate that small RNA pathways mediated by multiple Argonautes are required for optimal sperm production in males.

Although this study focuses on male fertility, we also assessed whether miRNA-associated Argonautes showed similar interactions in the regulation of hermaphrodite fertility. We therefore analyzed the production of self-progeny in our set of Argonaute mutants (Supplementary Fig. 2b and Table 4). All 3 primarily miRNA-associated Argonautes (*alg-1*, *alg-2* and *alg-5*) are required for optimal hermaphrodite fertility and fecundity. Interestingly, *alg-5; alg-3* and *alg-5; alg-4* double mutants displayed intermediate fertility compared to the corresponding single mutants (Supplementary Fig. 2b and Table 4). Because *alg-3*, *alg-4*, and *alg-5* regulate spermatogenesis in hermaphrodites, this result supports a genetic interaction between *alg-5* and *alg-3/alg-4*, likely mediated through miRNAs or their downstream targets. The stronger fecundity defects observed in multiply mutant hermaphrodites may result from additive effects of defects in both spermatogenesis and oogenesis.

### Male germline development depends on *alg-1* and *alg-2*

**Fig. 6.**
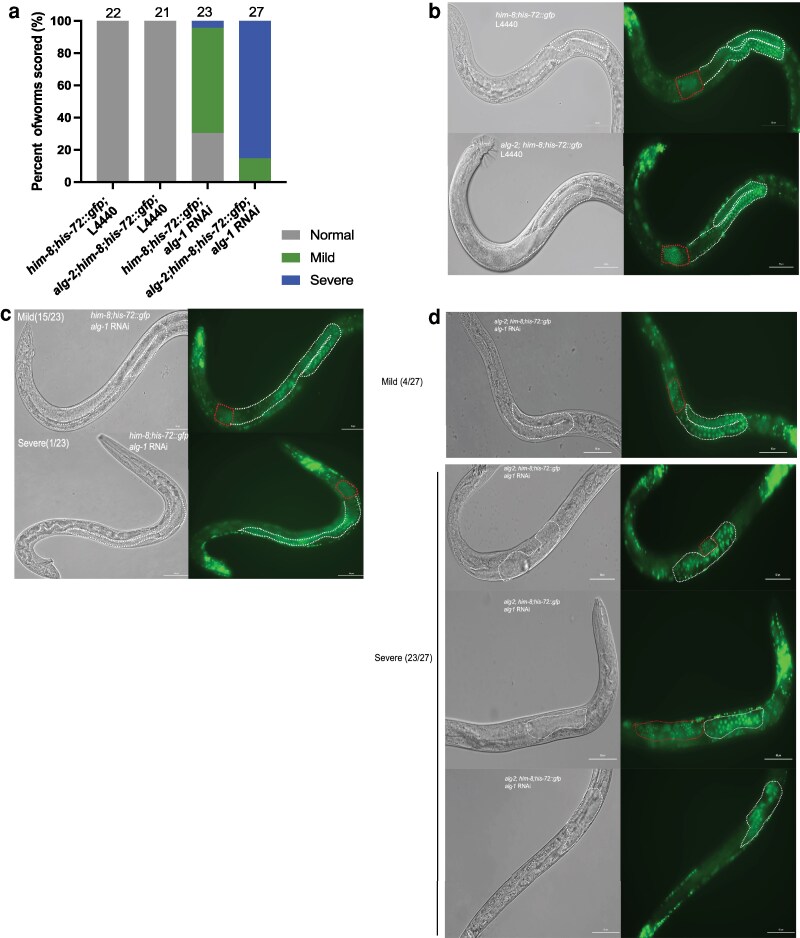
*alg-1* and *alg-2* function to regulate male germline development. a) Quantification of normal (gray) or defective gonad morphology (green, mild defects and blue, severe defects) in control or mutant males. The number of worms observed is indicated above each bar. b) Representative images of gonad morphology of control or *alg-2* mutant males when treated with control RNAi (L4440). c) Mild defects were observed in gonad morphology in *him-8;his-72::gfp* males treated with *alg-1* RNAi, displaying an extended localization of sperm in the proximal end of gonads. d) Severe gonad morphology defects are observed in *alg-2; him-8; his-72* males treated with *alg-1* RNAi. Few if any sperm (bottom) were observed and germline development and organization were strongly affected. b to d) A single focal plane from the Z-stack is shown, with DIC on the left and GFP on the right. The worm is positioned with its anterior end to the right of each image. The distal male gonad and more proximal condensed spermatids are outlined in white and red dashed areas, respectively. The scale bar = 50 μm.

ALG-1 and ALG-2 Argonaute proteins primarily bind miRNAs (Vasquez-Rifo et al. 2012; Seroussi et al. 2023) and have largely overlapping function in the soma. *alg-2*, but not *alg-1*, is expressed in the germ line (Seroussi et al. 2023). Because *alg-2; alg-1* double mutants are sterile (Grishok et al. 2001), we analyzed the effect of knocking down *alg-1* using RNAi in *alg-2* mutant worms. *alg-1* was knocked down in control and *alg-2* mutant males during larval development and young adult males were examined for germline defects. Both control and *alg-2* mutant males exhibited normal gonad morphology and meiotic progression from the distal to the proximal end and produced a large number of sperm under control RNAi conditions (L4440; Fig. 6b). In contrast, control males on *alg-1* RNAi had mild germline defects, including an extended proximal region or disorganized germ line, resulting in sperm localization in the anterior region (Fig. 6c). Control males treated with *alg-1* RNAi mostly display mild defects in gonad morphology (Fig. 6c), compared to worms treated with the negative control RNAi bacteria (Fig. 6, a and b). However, *alg-2* mutant males on *alg-1* RNAi had strong defects in germline development and organization (Fig. 6c), which resulted in little to no sperm production (Fig. 6d). Together, this indicates a requirement for *alg-1* and *alg-2* to support normal germline development, organization, and function.

## Discussion

Small RNA pathways are necessary for the proper regulation of gene expression for a wide range of biological processes. We performed phenotypic analysis to determine effects on male fertility and fecundity upon loss or reduced activity of genes in small RNA pathways that are known to be involved in miRNA biogenesis and miRNA activity, including Microprocessor genes Pasha (*pash-1*) and Drosha (*drsh-1*), miRNA-associated Argonaute genes (*alg-1*, *alg-2*, and *alg-5*), and miRNA and 26G-RNA associated Argonaute genes (*alg-3*, *alg-4*, and *rde-1)*. We determined that disruption of Microprocessor activity affected the ability of mutant males to produce cross-progeny, which requires both germline and somatic development and physiology to allow for normal mating behavior along with production and transfer of functional sperm capable of outcompeting hermaphrodite self-sperm to fertilize oocytes (Barr 2014). Our analysis revealed that small RNA pathway genes are necessary for optimal male fertility and fecundity, potentially functioning in the soma, in the germ line, or both.

It remains possible that defects observed in *pash-1*, *drsh-1*, and Argonaute family mutants are due to miRNA-independent functions. Drosha has been shown to have non-canonical roles, potentially able to bind mRNAs to function as a transcriptional activator or to promote cleavage and destabilization (Lee and Shin 2018). Similarly, Pasha/DGCR8 can interact with a range of RNA targets including mRNAs, snoRNAs, and long noncoding RNAs (Macias et al. 2012). Genetic evidence in *Drosophila melanogaster* also supports a Drosha-independent function for Pasha/DGCR8 (Luhur et al. 2014). While ALG-1 and ALG-2 interact nearly exclusively with miRNAs in *C. elegans*, ALG-3, ALG-4, ALG-5, and RDE-1 interact with 26G-RNAs along with miRNAs (Seroussi et al. 2023). It is expected that the miRNA-independent functions of Microprocessor genes and Argonaute family genes are independent of each other. Therefore, the shared defects in male fertility and sperm production are consistent with a model that these processes may be regulated by miRNA activity.

### Male fertility requires small RNA pathway genes in both soma and germline cells

We found that the production of cross-progeny by males was dependent on Microprocessor genes (*pash-1* and *drsh-1*) and a subset of Argonaute genes (*alg-1*, *alg-2*, *alg-3*, and *alg-5*). Using the *mj100* temperature sensitive allele of *pash-1*, we observed a failure of cross-progeny production by mutant males even at the permissive temperature of 15 °C, conditions at which 6% of mutant hermaphrodites show a gapped alae phenotype consistent with only a weak reduction of Microprocessor activity (Lehrbach et al. 2012). Additionally, endogenous miRNAs were detected at or near wild-type levels at 20 °C in *pash-1(mj100)* mutants (Lehrbach et al. 2012). Thus, a failure of *pash-1(mj100)* mutant males to produce cross-progeny at 15 °C and 20 °C suggests that male fertility is sensitive to even a weak reduction in Microprocessor activity. Further, expression of *pash-1(+)* from the *mjEx331* extrachromosomal array only partially rescued the ability of *pash-1(mj100)* mutant males to successfully produce cross-progeny at all temperatures examined. It is expected that somatic *pash-1* activity is restored in the presence of the *mjEx331* array, which expresses *pash-1* from the *eft-3* ubiquitous promoter, while germline *pash-1* activity would not be rescued due to the germline silencing of extrachromosomal arrays. Observed male fertility defects of *pash-1(mj100); mjEx331* males were enhanced at 25 °C compared to 15 °C, likely due to a further disrupted Microprocessor function in the germ line. Male fertility defects observed in the presence of *mjEx331* suggests that Microprocessor activity in the germ line may affect male fertility and the ability to sire cross-progeny. However, it remains possible that the reduced ability of *pash-1(mj100); mjEx331* males to sire cross-progeny is due to the incomplete rescue of *pash-1* activity in the soma. In contrast, *pash-1(mj100); mjEx331* males that successfully mated were able to produce wild-type levels of cross-progeny at all temperatures, indicating that sperm activation and sperm function is not sensitive to reduced levels of Microprocessor activity. Similar to *pash-1* mutant analysis, *drsh-1* mutant males displayed severe defects in the ability to produce cross-progeny in mating assays. These *drsh-1* mutants were first-generation homozygous mutants isolated from heterozygous hermaphrodites that are viable and overall healthy (Denli et al. 2004). This also supports the model that male fertility is sensitive to modest reductions in Microprocessor activity and possibly miRNA function.

Furthermore, our analysis with Argonaute mutants supports a role for small RNA pathways in both the soma and the germ line to regulate male fertility and mating success. The ability to sire cross-progeny requires *alg-1* function, which is detected in the soma but not in the germ line. Loss of the germline specific Argonaute, *alg-5*, enhances defects of *alg-2* and *alg-3* mutants, suggesting small RNA activity in the germ line may impact male mating success. Surprisingly, loss of *alg-2*, *alg-3*, *alg-4*, and *alg-5* together was not associated with the reduced ability to sire cross-progeny, possibly due to complex genetic interactions of small RNA pathways. A few miRNAs have been identified as regulators of male fertility. *mir-57* and *mir-35-41* function to regulate male tail development (Zhao et al. 2010; McJunkin and Ambros 2014), while the *mir-35* family is also involved in the regulation of male mating (McJunkin and Ambros 2014). The roles of individual miRNAs and their targets in this process remain largely unknown. It remains to be determined whether the male fertility defects in these mutants is attributed to defects in male mate finding, male-hermaphrodite interactions during mating, or in sperm transfer. Collectively, this evidence supports an important role for small RNA pathway genes and miRNA-associated Argonautes in promoting male fertility.

### Male fecundity requires Microprocessor and Argonaute gene activity

Male fecundity requires optimal sperm production, sperm transfer, and sperm activation to generate cross-progeny upon successful mating with hermaphrodites. We observed that sperm production, but not sperm activation, is sensitive to *pash-1* disruption, as shown by fewer sperm at both 20 °C and 25 °C in *pash-1(mj100)* mutant males at the L4 molt stage. In contrast, *drsh-1* mutant males displayed a modest increase in sperm production, suggesting that a subset of miRNAs may negatively regulate sperm production. Alternatively, this difference between *drsh-1* and *pash-1* mutants may reflect miRNA-independent activity of *pash-1* in the process of sperm production. While loss of *alg-1* and *alg-2* was found to be associated with a reduced number of sperm in L4 molt stage males, even fewer sperm were observed in males missing both *alg-1* and *alg-5* activity. This defect likely is due in part to reduced miRNA function since multiple miRNAs have been identified to be necessary for sperm production in males (Lu and Abbott 2024).

Additional Argonautes are also necessary for male fecundity. ALG-3 and ALG-4 are known to regulate spermiogenesis through functioning with 26G-RNAs for the positive regulation of mRNA targets (Conine et al. 2010, 2013). However, these proteins also interact with miRNAs in the germ line (Seroussi et al. 2023). We observed genetic interactions that suggest shared pathway regulation between *alg-3* and *alg-4* with *alg-2* and *alg-5* for both the generation of cross-progeny and for sperm production. Matings with *alg-5; alg-2; alg-4; alg-3* mutant males resulted in a reduced number of cross-progeny compared to *alg-4; alg-3* double mutant males. While *alg-5; alg-2; alg-4; alg-3* had fewer sperm at the L4 molt stage compared to controls, males produced an even lower number of cross-progeny, suggesting additional defects in sperm transfer or sperm function. Similar results were observed with *drsh-1*/hT2 heterozygous males, suggesting a shared sensitivity to reduced small RNA pathway activity for sperm production, transfer, or function. This could reflect interactions with shared miRNAs or alternatively more indirect interactions with downstream gene targets. Collectively, these results suggest that miRNA regulation contributes to processes necessary for optimal male reproductive fitness.

## Supplementary Material

jkag079_Supplementary_Data

## Data Availability

The authors affirm that all data necessary for confirming the conclusions of the article are present within the article, figures, and tables. All strains analyzed in this paper are available upon request. Supplemental material available at G3 online.
