# Supplementary material for: Optimal male fertility and fecundity in Caenorhabditis elegans require Microprocessor and Argonaute gene function
Source: G3 (Bethesda). 2026 Apr 2;16(6):jkag079. doi: 10.1093/g3journal/jkag079 (PMC13232498; doi:10.1093/g3journal/jkag079)
Supplement: jkag079_Supplementary_Data [file jkag079_supplementary_data.zip › Supplemental_Material_G3-2026-406652.pdf]

Supplementary Material

Lu et al. "Optimal male fertility and fecundity in *Caenorhabditis elegans* requires Microprocessor and Argonaute gene function"

- Figure S1
- Figure S2

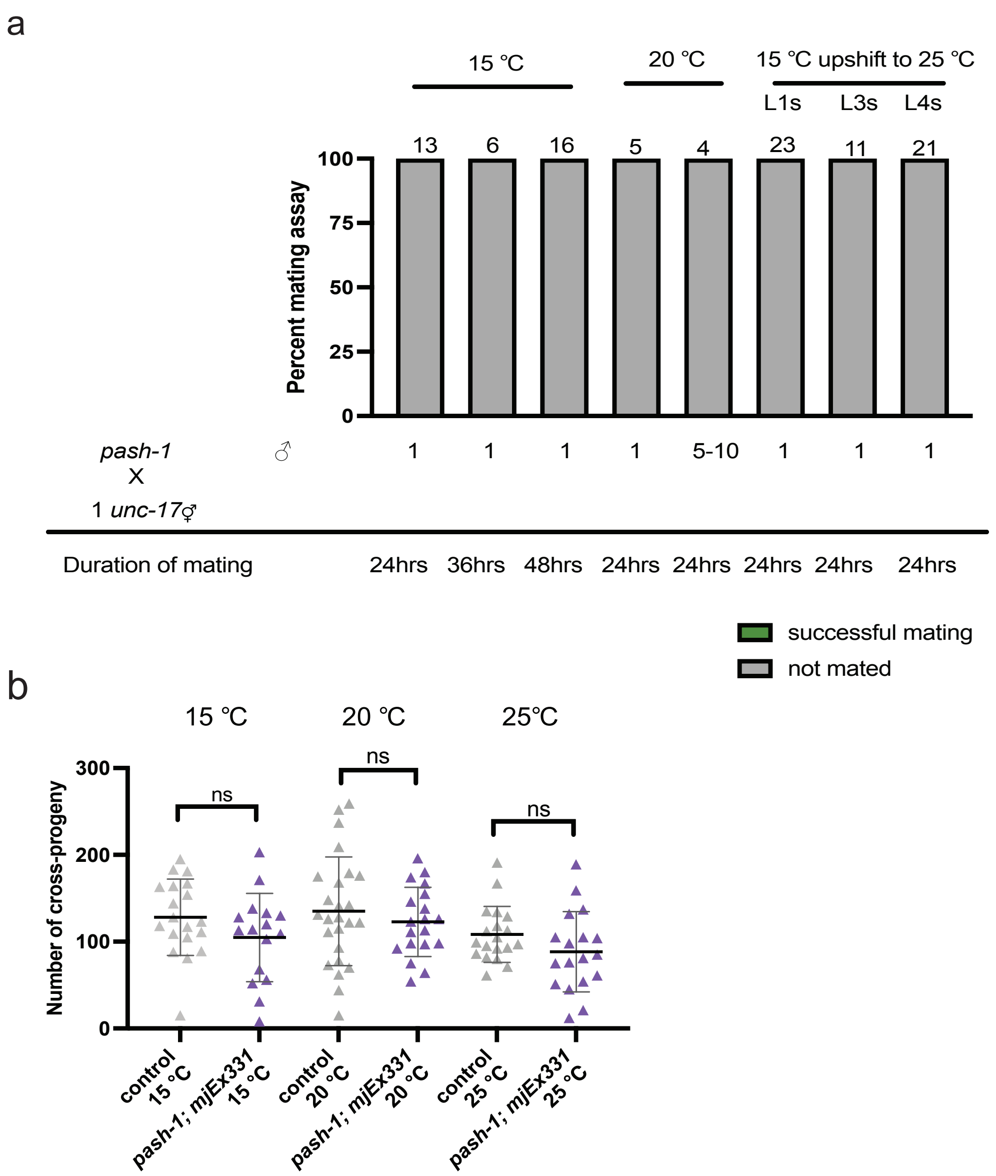

Fig. S1 *pash-1* mutants displayed male fertility defects. a) No mating assays resulted in the production of cross progeny for *pash-1* mutant males in assays with either a single male or multiple males at permissive or restrictive temperatures. The number of mating assays is indicated above each bar. b) *pash-1;mjEx331* males (purple) sired a comparable number of cross-progeny relative to control males (gray). Each triangle represents the number of cross-progeny from a successfully mated hermaphrodite, and lines indicate mean ± SD (b). Comparisons between control (*him-8; his-72::gfp*) and mutant cross progeny results were conducted using Welch's t-test.

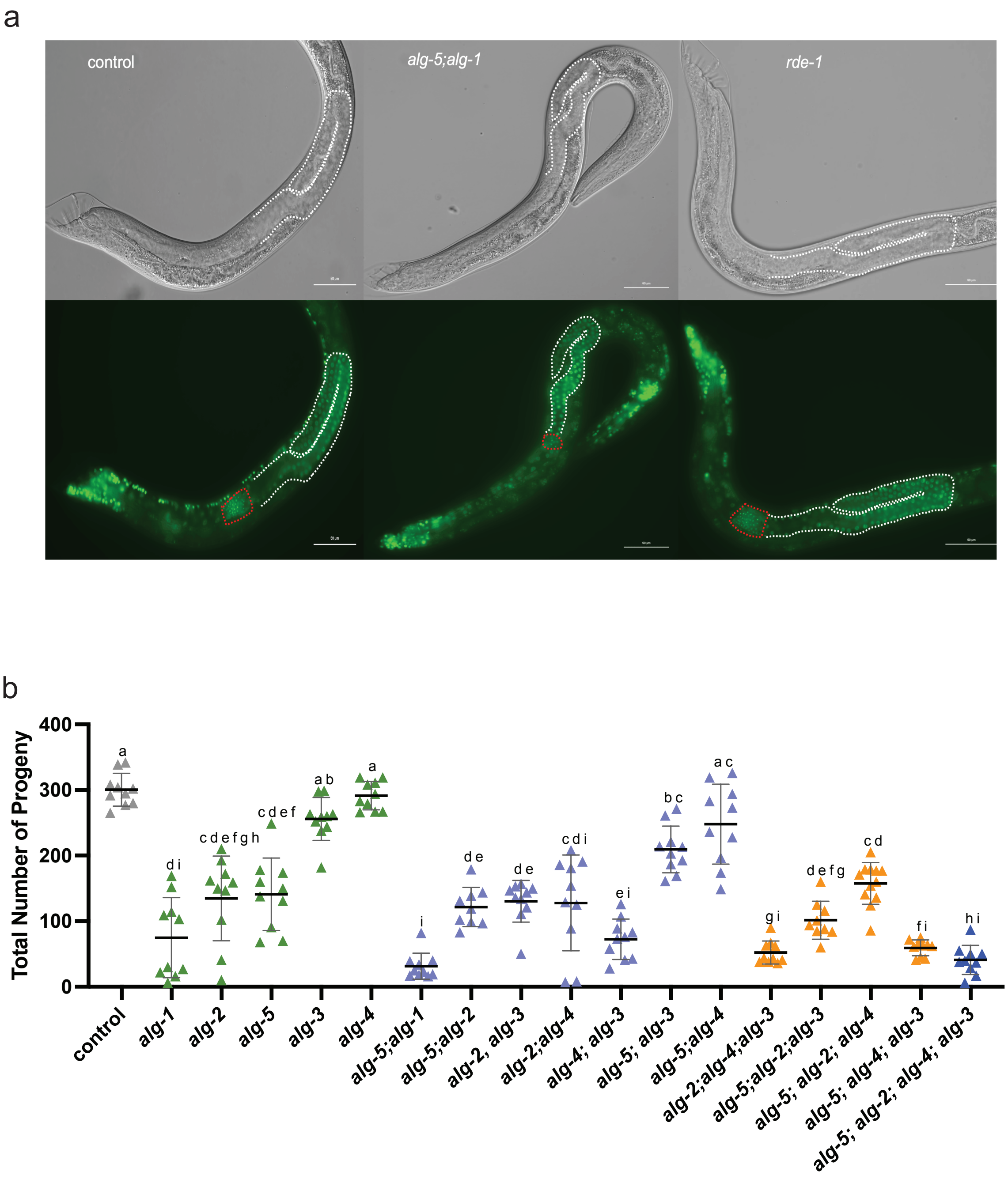

Fig. S2 Mutations in Argonaute genes affect male gonad morphology and hermaphrodite fertility. a) Representative male gonad of control or mutant worms (*alg-5; alg-1* and *rde-1*). Each worm is oriented with the tail on the left of each image. A single focal plane from the Z-stack is shown, with the distal gonad arm and proximal condensed spermatids outlined in white and red, respectively. n=13-17 males analyzed and the scale bar = 50 μm. b) Loss of individual and multiple Argonaute encoding genes results in significantly reduced brood size. Each dot represents an individual worm, and the error bar indicates mean ± SD. Comparisons were conducted using one-way ANOVA followed by Tukey's post hoc test, and statistical differences are denoted by letters. Different letters indicate statistically significant differences (p < 0.05), whereas groups sharing a letter are not significantly different.
